# Prostate brachytherapy: HDR or seed implant

**DOI:** 10.4103/0971-6203.29193

**Published:** 2006

**Authors:** K. R. Das

**Affiliations:** Peter MacCallum Cancer Centre, Melbourne 3002, Australia. E-mail: ram.das@petermac.org

“I crave the forgiveness of the learned for giving an interpretation different from the orthodox commentarians in regard to these two verses”.

Kathopanishad

Incidence of prostate cancer among men has been on the increase in India and as per many cancer registries it is sixth in incidence rate. This may be due to the increase in longevity among the population, increased awareness and the early detection with PSA testing. A number of treatment options are now available, which include watchful waiting, radical prostatectomy, external beam irradiation and brachytherapy. Of all these modalities, brachytherapy seems to be very promising for localised prostate cancer in terms of local control, patient convenience, morbidity and cost-effectiveness.

Prostate brachytherapy involves the technique of implanting discrete radiation source either permanently or temporarily in the prostate. Both techniques are used either in conjunction with external beam radiotherapy or as monotherapy. However, ^192^Ir HDR brachytherapy is usually used to deliver a boost to external beam radiotherapy while ^125^I seed implants are more commonly used as single modality treatment, restricting its application to early stage prostate cancer. It is notable that different groups in India are working on both these techniques and dosimetry.[1,2] With the technological advancement in ultrasound equipment, fixing devices, US probe carriers, treatment planning systems and the availability of a variety of source configurations, brachytherapy is the choice of many physicians and even more importantly patients.

However, the success of any chosen modality depends upon accurate patient selection using, PSA level, Gleason Score, pathological staging and Partin's Table.

Initial trials of implanting radioactive seeds of ^125^I through open retropubic route had resulted in poor results in localised prostate cancer.[3] Transperineal percutaneous implants were introduced by Kumar[4] for ^125^I seeds and by Miller[5] for ^192^Ir wire. Since then the transperineal approach with trans-rectal ultrasound guidance has vastly improved the results.[6] In the late 1980s Martinez in USA and Bertermann in the Netherlands started to use the same technique for HDR brachytherapy with ^192^Ir source. Currently HDR monotherapy and seed implants with ^125^I, ^103^Pd and ^131^Cs are extensively used in management of prostate cancer.

There are extensive discussions on the merits and demerits of seed implant therapy and HDR monotherapy. It is generally accepted that seed implant will not be suitable for prostate of volume more than 40 cm^3^ and for clinical staging beyond T2b, whereas prostate of larger volumes and staging up to T3c can be treated with HDR monotherapy. Another restriction in seed therapy is the interference of the pubic arch, which does not allow the placing of the seeds beyond the arch. It is also generally accepted that seeds should not be implanted in patients who had TURP done less than 6 months prior.

The single stepping source with variable dwell times used in HDR monotherapy has the greater potential of creating an optimised treatment plan after placement of the needles. Further, any needle movements could be accounted for in modifying the plan before subsequent fractions. However with seed implants seed migration might be a problem seed migration within the prostate and its periphery, both of which alter the planned dose distribution.[7] There have been reports of seeds embolized in lung.[8,9] This problem has been greatly reduced[10] with the use RAPID Strands^®^ and similar approaches where multiple seeds are embedded in a plastic strand reducing the risk of movement. RAPID Strand^®^ also has helped improve the target coverage compared to use of individual seeds as seed implantation without migration outside of the prostate capsule is possible.[11]

Currently in the management of prostate cancer the whole prostate with any extracapsular extension is treated though mostly the cancer is confined to the peripheral zones. Hence the prescription is typically to a minimum peripheral dose on the target volume for both techniques. The dose is 145 Gy for ^125^I seed implants and 115 Gy for ^103^Pd seed implants as per the recommendations of the American Association of Physicists in Medicine (AAPM) task group (TG 43) and guidelines of the American Brachytherapy Society (ABS).[12,13] However, no such recommendations exist for HDR brachytherapy yet. The number of HDR fractions and the fraction sizes are still debatable and vary widely from institution to institution. Clinical trials by various groups employ various fraction schedules. The William Beaumont group employs a total dose of 38 Gy delivered in four fractions over two days.[14] The Osaka group uses 48 Gy in 8 fractions or 54 Gy in nine fractions, with two fractions per day.[15] In dose escalation studies, Martinez and colleagues[16] have used two fractions with 11.5 Gy per fraction. Most fractionation schedules are worked out to give an effective dose of more than 90 Gy @ 2 Gy fractions with the assumption that the α/β ratio is about 2.3 and the repair half time is 1.5 h. These values are still being debated[17] as to their validity for irradiation with low dose rate for months compared to multiple fractions at high dose rate delivered within a couple of days. However it is generally accepted that prostate cancer with an α/β ratio less than 3 is more responsive to hypofractionated HDR treatment.[18]

The dosimetry of seed implants may be a little questionable. Generally a preplan is generated based on ultrasound volume study performed 3-4 weeks prior to the implant. The DVH for D_90_ (the dose delivered to 90% of the target volume) 30 days after the implant, is the golden parameter. It is assumed that by then the swelling of the gland due to the trauma of the implant has ceded and no more seed movement will take place. However since only about 30% of the prescription dose is delivered by 30^th^ day (for ^125^I implants) and the possibility of seed movements still later, it is surprising that the D_90_ on Day_30_ is accepted as a standard for the implant. Worse still, if one assumes that prostate cancer is slow growing, theoretically the delivery of the prescription dose over about 10 months, as with ^125^I and ^103^Pd may have an adverse effect on tumor control[19] since the dose rate falls well below 0.2Gy per day, the sterilizing dose for slow growing tumors.[20]

However, there is no doubt that considerable clinical experience has accumulated within the last 15 years with ^125^I seed therapy whereas ^103^Pd or ^131^Cs seed therapy is relatively new. On the other side it is only within the last 5 years that HDR monotherapy has been initiated. Hence it is still too early for a comparison of clinical outcomes. Both the techniques are similar with regards to the use of trans rectal ultrasound. However with post implant dosimetry and optimization methods with a single source, HDR dosimetry is more flexible and accurate. Battermann and colleagues[21] have summarised the clinical results of ^125^I seed implant. With a mean follow-up of 50 months (24-123 months) of 351 patients, they report an actuarial survival of 85% at 5 years and 76% at 7 years while actuarial bNED estimated freedom at 5 years was 64% and at 7 years 51%. With HDR monotherapy, a 3-year local control, overall survival and PSA failure free rates have been reported as 100, 97 and 83% with 111 patients with a media FU of 27 months, range 5-119 months.[15] The corresponding 5 year rates are 97, 92 and 70%. A randomized prospective trial with a head to head comparison is still outstanding.

But local control is not the whole story. Generally the urinary complications reported are higher with seed implants. Grills[14] reported decreased acute rates of grade 1-3 dysuria with HDR monotherapy (36%) against 67% (*P*<0.001) for ^103^Pd seed implant.. They also noted a reduction in urinary frequency 92% *vs*. 54% for HDR and LDR techniques (*P*<0.001). Though urethral stricture of 8% was reported for the HDR group against 3% for LDR group (*P*=0.177), 3-year actuarial impotence rate was only 16% in the HDR group, against 45% for the LDR group. The clinical results cannot be reliably compared as the HDR patients are generally in the higher risk group with increased PSA and advanced staging.

Finally, considerations must be given to resource requirements. Once the HDR unit is installed, the total cost including source changes can be shared with all HDR treatments. However, the cost of seeds varying in number from 80 to 120, will have to be charged to individual patients. Financial studies have shown that if more than 20-30 patients are treated annually, HDR monotherapy is more economical than seed therapy.[22] Since the HDR treatments are given only in appropriately shielded rooms the patient does not harbour any radioactivity outside the room. However, with permanent implants, the seeds are left in the patient allowing to decay with their inherent half-life. Though the dose levels around the patient are low, this may still be of concern for children and pregnant women at home. When comparing these two modalities, the clinical outcome depends upon the selection of patients, conformal dosimetry, correct fractionation and total dose, local control, toxicities, patient comfort and acceptability and cost. A comparison of these parameters is given in Table 1.

**Table 1:** Comparison of high dose rate temporary implants (monotherapy) and permanent seed implants

	*HDR*	*Seed implant*
Conformal treatment	Excellent	Very good
Target accuracy	Excellent	Very good
Ability to treat extra-capsular extension	Very good	Fair
Ability to treat seminal vesicles	Very good	Good
Ease of control of radiation	Excellant	Good
Lack of cold/hot spots	Very good	Good
Control of critical organ dose	Very good	Good
Modify dose distribution	Excellent	Fair
Need for external beam	No	Sometimes
Experience of physician	Crucial	Crucial
Pre-planning	No	Extensive
Post-implant dosimetry	No	Extensive (CT)
Stages treated	T1–T3	T1–T2
Gland volume >50 cm^3^	Yes	Can't be done
Pubic arch interference	Workable	Can't be done
Prior turp	Less problem	More problem
Final dose verification	Pre-treatment	Post-treatment
Symptom duration	Weeks	Months
Source/needle movements	Possible*	Migration**
Number of visits to hospital	One***	At least 3
Need for hospitalization	Two days	At least one day
Cost	Decreases with case load	High
Radiation exposure to public	Practically NIL	Possible

* Needles may move in-between fractions, but could be re-imaged and replanned before treatment.

** Less chance with RAPID Strands^â^

*** Two visits in some hospitals performing two separate implants

In conclusion, HDR brachytherapy for prostate cancer can be delivered more conformally and safely with reduction in overall cost. Patients tolerate the technique very well. Results of long-term follow-up need to be awaited, however, one can expect them to be quite favorable for this modality. In fact, the trend in USA indicates an increased rate in the use of HDR brachytherapy relative to seed implants (Nissar Syed, Personal communication 2006).
